# Soulamarin, a New Coumarin from Stem Bark of *Calophyllum soulattri*

**DOI:** 10.3390/molecules16119721

**Published:** 2011-11-23

**Authors:** Gwendoline Cheng Lian Ee, Siau Hui Mah, Soek Sin Teh, Mawardi Rahmani, Rusea Go, Yun Hin Taufiq-Yap

**Affiliations:** 1 Department of Chemistry, Faculty of Science, University Putra Malaysia, 43400 UPM Serdang, Selangor, Malaysia; 2 Department of Biology, Faculty of Science, University Putra Malaysia, 43400 UPM Serdang, Selangor, Malaysia

**Keywords:** soulamarin, pyranocoumarin, *Calophyllum soulattri*, Clusiaceae

## Abstract

The extracts of the stem bark of *Calophyllum soulattri* gave a new pyranocoumarin, soulamarin (**1**), together with five other xanthones caloxanthone B (**2**), caloxanthone C (**3**), macluraxanthone (**4**), trapezifolixanthone (**5**) and brasixanthone B (**6**) one common triterpene, friedelin (**7**), and the steroidal triterpene stigmasterol (**8**). The structures of these compounds were established based on spectral evidence (1D and 2D NMR).

## 1. Introduction

The genus *Calophyllum* comprises over 200 species of trees and shrubs native to tropical Asia, East Africa, India and Australia. *Calophyllum* species have been used in traditional Chinese folk medicine for the treatment of wounds, inflammation and rheumatism. Phytochemical studies on *Calophyllum* species have revealed the presence of xanthones [1,2,3], coumarins [4], triterpenoids [5] and flavonoids [6]. The coumarins have been reported to have displayed anti-HIV biological effects [7] and are used as cancer chemo-preventive agents [8]. Various xanthone derivatives show antifungal [9], antimicrobial [10] and molluscicidal [11] effects. We report here the isolation and characterization of a new pyranocoumarin, soulamarin (**1**), from the stem bark of *Calophyllum soulattri.*

## 2. Results and Discussion

Soulamarin (**1**) was isolated as yellowish oil from the hexane extract of the stem bark of *Calophyllum soulattri*. The HRESIMS displayed a negative molecular ion peak at *m/z* 387.1818 [M−H]^−^ indicating a molecular formula C_22_H_28_O_6_. Compound **1** gave typical coumarin IR absorptions at 3,296 (OH), 2,930 (sp^2^ and sp^3^ CH), 1,706 (C=O) and 1,621 (C=C) cm^−1^. Maximum absorptions were observed at 314, 300, 274 and 268 nm in the UV spectrum [12].

The ^1^H-NMR spectrum revealed the presence of one chelated hydroxyl proton signal at δ 12.46 (*s*, 1H), two vinylic proton signals at δ 6.60 (*d*, 1H, *J* = 10.1 Hz) and 5.46 (*d*, 1H, *J* = 10.1 Hz,) for H-9 and H-10 respectively, three methine signals at δ 2.53 (*m*, H-2'), 4.09 (*m*, H-3') and 3.80 (*m*, H-4) and three methylene signals at δ 1.26 (*m*, H-14), 1.38 (*m*, H-15) and 2.71 and 2.81 (both *dd*, *J* = 7.8 Hz) for H-3a and H-3b. Five methyl signals at δ 1.19 (*d*, *J* = 6.4 Hz), 1.27 (*t*, *J* = 3.4 Hz), 1.42 (*s*), 1.44 (*s*) and 1.49 (*d*, *J* = 6.4 Hz) were also observed. The^ 13^C-NMR spectrum and DEPT experiment showed the presence of seven quaternary carbons at δ 78.2 (C-11), 102.1 (C-5), 102.9 (C-7), 110.9 (C-4a), 157.0 (C-6), 159.6 (C-8) and 159.7 (C-8a), five methines at δ 25.4 (C-4), 45.8 (C-2'), 79.0 (C-3'), 115.8 (C-9), 125.8 (C-10), three methylenes (δ 39.5, 28.4, 29.8 for C-3, C-14 and C-15, respectively) and five methyls (δ 10.4, 19.3, 19.7, 28.3 × 2 for C-5', C-16, C-4' and C-12 and C-13, respectively). The ^13^C-NMR spectrum also indicated a carbonyl (δ 179.1) and an aliphatic ketone (δ 199.4).

**Figure 1:**
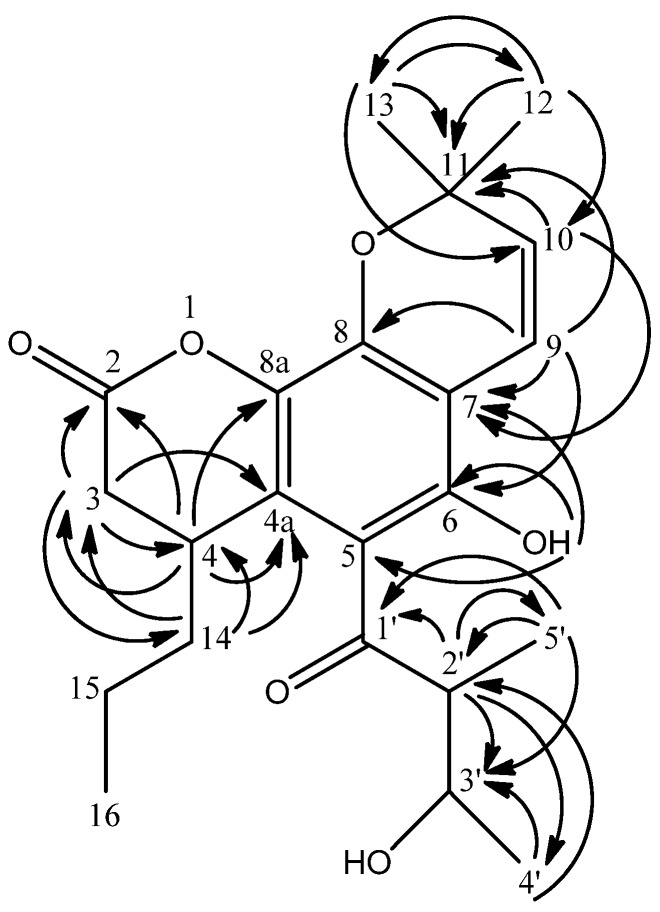
HMBC ^2^*J* and ^3^*J* correlations between ^1^H and ^13^C in **1**.

The structure was further elucidated by HMBC spectral analysis after the assignment of the protons to their direct bonding carbons by the HMQC spectrum. The low field chelated proton at δ 12.46 was due to hydrogen-bonding with the carbonyl group (δ 199.4, C=O) which resulted in the deshielding effect. The ^3^*J* and ^2^*J* connectivity of the chelated hydroxyl proton at δ 12.46 with δ 102.1 (C-5), 102.9 (C-7) and 157.0 (C-6) confirmed its location at C-6 (see Figure 1).

The HMBC experiment also demonstrated long-range ^2^*J* and ^3^*J* correlations between the two doublet protons at δ 5.46 (H-10, *J* = 10.1 Hz) and 6.60 (H-9, *J* = 10.1 Hz) with the carbon signal at δ 78.2 (C-11) respectively. The linkages between the two aliphatic methyls at δ 1.42 (H-12) and δ 1.44 (H-13) to the two carbon signals at δ 78.2 (C-11) and 125.8 (C-10) were also seen. These data together with the COSY spectrum analysis suggested the existence of a pyran ring. The pyran ring was clearly fused onto the non-oxygenated carbon C-7 and an oxygenated carbon C-8, as confirmed by the long range (^3^*J)* correlations of H-9 (δ 6.60) with C-8 (δ 159.6) and H-10 (δ 5.46) with C-7 (δ 102.9).

Meanwhile, the HMBC correlations of δ 1.19 (H-5') with δ 45.8 (C-2'), 79.0 (C-3') and 199.4 (C-1') proved that these methyl protons were located at position C-2'. The coupling of H-2' and H-3' in the COSY spectrum established the connectivity between δ 45.8 (C-2') and 79.0 (C-3'). The linkage between δ 1.49 (H-4') with δ 45.8 (C-2') and 79.0 (C-3') confirmed its position at C-4'.

**Figure 2:**
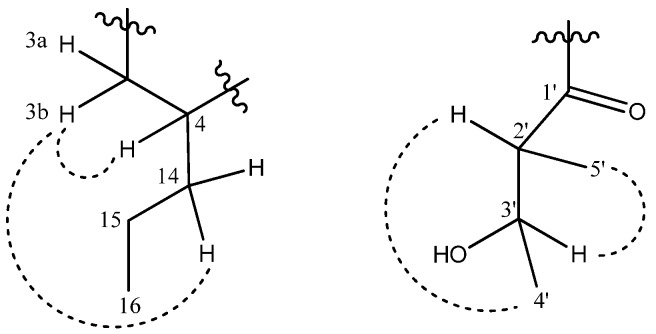
NOESY correlations between ^1^H and ^1^H in **1**.

**Figure 3:**
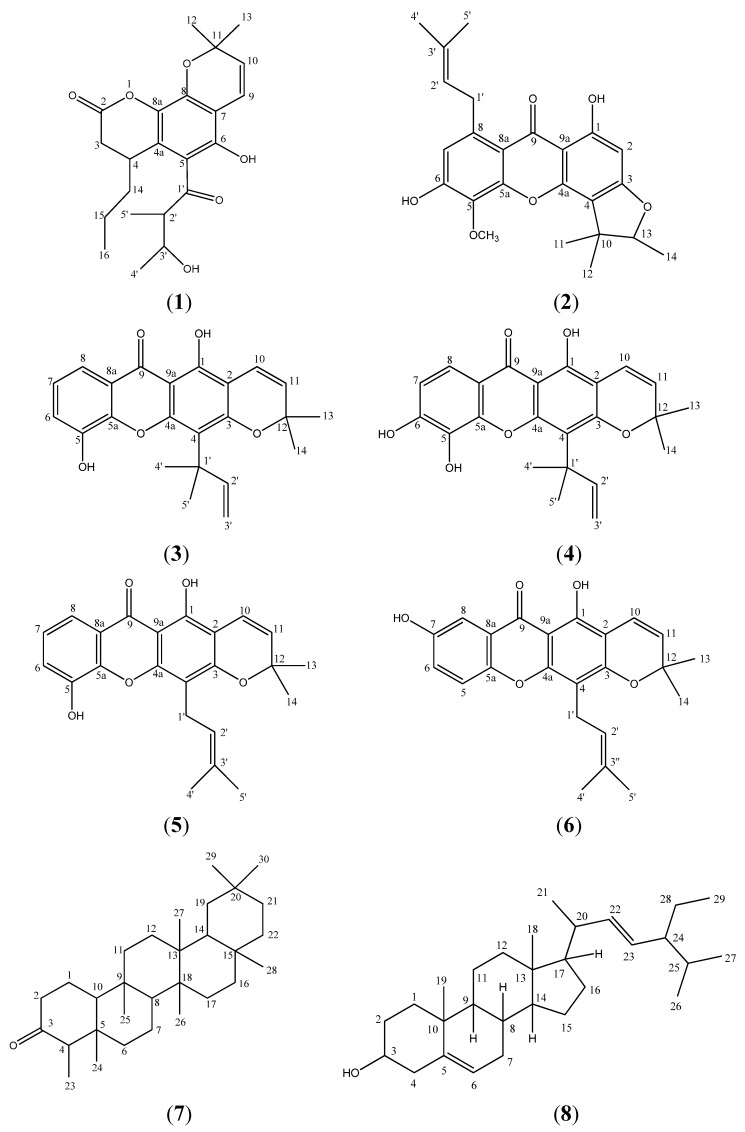
Structures of soulamarin (**1**), caloxanthone B (**2**), caloxanthone C (**3**), macluraxanthone (**4**), trapezifolixanthone (**5**), brasixanthone B (**6**), friedelin (**7**) and stigmasterol (**8**).

An *n*-propyl substituent was found to be attached to C-4 via long range correlations of δ 1.26 (H-14) with δ 25.4 (C-4), 39.5 (C-3) and 110.9 (C-4a). The existence of the *n*-propyl group was also observed in the COSY spectrum through cross peaks between δ 1.26 (H-14) and 1.38 (H-15) and between δ 1.38 (H-15) and 1.27 (H-16). NOESY experiments predicted the relative configurations of soulamarin. Cross peaks were observed between H-3' and H-5' and between H-2' and H-4' suggesting the two proton pairs were oriented on opposite sides. Cross peaks between H-3b and H-4 and between H-3b and H-14 indicate these protons have similar orientations. The NOESY correlations for compound **1** are shown in Figure 2. Taken together, the evidence suggested that compound **1** is 6-hydroxy-4-propyl-5-(3-hydroxy-2-methyl-1-oxobutyl)-6",6"-dimethylpyrano-[2",3":8,7]-benzopyran-2-one (Figure 3).

## 3. Experimental

### 3.1. Plant Material

The stem bark of *Calophyllum soulattri* was collected from the Sri Aman district in Sarawak, Malaysia. This plant was identified by Dr. Rusea Go from the Department of Biology, Faculty of Science, Universiti Putra Malaysia.

### 3.2. General

EIMS were recorded on a Shimadzu GC-MS model QP2010 Plus spectrometer. NMR spectra were obtained using a JEOL 500 MHz FT-NMR spectrometer using tetramethylsilane (TMS) as an internal standard. Ultraviolet spectra were recorded in EtOH on a Shimadzu UV-160A, UV-Visible Recording Spectrophotometer. Infrared spectra were measured using the universal attenuated total reflection (UATR) technique on a Perkin-Elmer 100 Series FT-IR spectrometer.

### 3.3. Extraction and Isolation

Approximately 1 kg of air-dried stem bark of *Calophyllum soulattri* was ground to a fine powder and extracted successively in a Soxhlet apparatus with *n*-hexane (68-72 °C, 3 × 2 L) and dichloromethane (40 °C, 3 × 2 L) for 24 hours. The extracts were evaporated to dryness under vacuum to give 101.2 g of *n*-hexane extract and 15.3 g of dichloromethane extract. Part of each extract was subjected to column chromatography over silica gel and eluted with a stepwise gradient system of *n*-hexane, dichloromethane, ethyl acetate and methanol. Further purification of the *n*-hexane extract afforded the new coumarin soulamarin (**1**, 8 mg) and the triterpene friedelin (7, 450 mg). Compound **1** was isolated from the *n*-hexane:chloroform (1:4) eluate mixture, followed by several further purifications using a Chromatotron® (Harrison Research) eluting with an *n*-hexane:chloroform (3:2) mixture. *Soulamarin* (**1**): Yellowish Oil; UV (EtOH) λ_max_ nm: 314, 300, 274, 268; IR *ν_max_* cm^−1^: 3296, 2930, 1706, 1621; EIMS *m*/*z* (rel. int.): 388 [M^+^] (9), 373 (53), 360 (42), 345 (100), 301 (14), 285 (13), 245 (9), 229 (24), 55 (11); HRESIMS: 387.1818 [M-H]^−^ (Calc’d. for C_22_H_28_O_6_: 388.1886); ^1^H-NMR (CDCl_3_): *δ* 12.46 (OH-6, s), 6.60 (1H, *d*, *J* = 10.1 Hz, H-9), 5.46 (1H, *d*, *J* = 10.1 Hz, H-10), 4.09 (1H, *m*, H-3'), 3.80 (1H, *m*, H-4), 2.81 (1H, *dd*, *J* = 7.8 Hz, H-3a), 2.71 (1H, *dd*, *J* = 7.8 Hz, H-3b), 2.53 (1H, *m*, H-2'), 1.49 (3H, *d*, *J* = 6.4 Hz, H-4'), 1.44 (3H, *s*, H-13), 1.42 (3H, *s*, H-12), 1.38 (2H, *m*, H-15), 1.27 (3H, *t*, *J* = 3.4 Hz, H-16), 1.26 (2H, *m*, H-14), 1.19 (3H, *d*, *J* = 6.4 Hz, H-5'); ^13^C-NMR (CDCl_3_): *δ* 199.4 (C-1'), 179.1 (C-2), 159.7 (C-8a), 159.6 (C-8), 157.0 (C-6), 125.8 (C-10), 115.8 (C-9), 110.9 (C-4a), 102.9 (C-7), 102.1 (C-5), 79.0 (C-3'), 78.2 (C-11), 45.8 (C-2'), 39.5 (C-3), 29.8 (C-15), 28.3 × 2 (C-12 & C-13), 25.4 (C-4), 19.7 (C-4'), 19.3 (C-14 & C-16), 10.4 (C-5'). Meanwhile, purification of the dichloromethane extract afforded the five known xanthones caloxanthone B (**2**, 12 mg), caloxanthone C (**3**, 14 mg), macluraxanthone (**4**, 6 mg), trapezifolixanthone (**5**, 10 mg), brasixanthone B (**6**, 21 mg) and stigmasterol (**8**, 22 mg) (Figure 3).

*Caloxanthone B* (**2**). Yellow needles; M.P. 157–158 °C (lit. 160.5 °C [13]); Spectral data were consistent with published data [13].
*Caloxanthone C* (**3**). Yellow needles; M.P. 210–212 °C (lit. 217 °C [14]); Spectral data were consistent with published data [14].
*Macluraxanthone* (**4**). Yellow crystal; M.P. 174–175 °C (lit. 170–172 °C [13]); Spectral data were consistent with published data [13].
*Trapezifolixanthone* (**5**). Yellow crystal; M.P. 171–172 °C (lit. 171–172 °C [15]); Spectral data were consistent with published data [15].
*Brasixanthone B* (**6**). Yellow crystal; M.P. 227–229 °C (lit. 227–229 °C [16]); Spectral data were consistent with published data [16].
*Friedelin* (**7**). White needles; M.P. 245–246 °C (lit. 246–248 °C [2]); Spectral data were consistent with published data [2].
*Stigmasterol* (**8**). White needles; M.P. 155–157 °C (lit. 168–169 °C [17]); Spectral data were consistent with literature data [17].

## 4. Conclusions

The stem bark of *Calophyllum soulattri* furnished one new pyranocoumarin, soulamarin (**1**), together with five xanthones caloxanthone B (**2**), caloxanthone C (**3**), macluraxanthone (**4**), trapezifolixanthone (**5**) and brasixanthone (**6**), a common triterpene, friedelin (**7**), and the steroidal triterpene stigmasterol (**8**).
